# Decreased Peripheral and Central Responses to Acupuncture Stimulation following Modification of Body Ownership

**DOI:** 10.1371/journal.pone.0109489

**Published:** 2014-10-06

**Authors:** Younbyoung Chae, In-Seon Lee, Won-Mo Jung, Dong-Seon Chang, Vitaly Napadow, Hyejung Lee, Hi-Joon Park, Christian Wallraven

**Affiliations:** 1 Department of Brain and Cognitive Engineering, Korea University, Seoul, Korea; 2 Acupuncture and Meridian Science Research Center, College of Korean Medicine, Kyung Hee University, Seoul, Korea; 3 Department of Human Perception, Cognition and Action, Max Planck Institute for Biological Cybernetics, Tübingen, Germany; 4 Martinos Center for Biomedical Imaging, Massachusetts General Hospital, Harvard Medical School, Charlestown, Massachusetts, United States of America; 5 Department of Biomedical Engineering, Kyunghee University, Yongin, Korea; University G. d'Annunzio, Italy

## Abstract

Acupuncture stimulation increases local blood flow around the site of stimulation and induces signal changes in brain regions related to the body matrix. The rubber hand illusion (RHI) is an experimental paradigm that manipulates important aspects of bodily self-awareness. The present study aimed to investigate how modifications of body ownership using the RHI affect local blood flow and cerebral responses during acupuncture needle stimulation. During the RHI, acupuncture needle stimulation was applied to the real left hand while measuring blood microcirculation with a LASER Doppler imager (Experiment 1, *N* = 28) and concurrent brain signal changes using functional magnetic resonance imaging (fMRI; Experiment 2, *N* = 17). When the body ownership of participants was altered by the RHI, acupuncture stimulation resulted in a significantly lower increase in local blood flow (Experiment 1), and significantly less brain activation was detected in the right insula (Experiment 2). This study found changes in both local blood flow and brain responses during acupuncture needle stimulation following modification of body ownership. These findings suggest that physiological responses during acupuncture stimulation can be influenced by the modification of body ownership.

## Introduction

The importance of an accurate representation of the body by the brain has been documented in many different studies involving both healthy participants and clinical patients [1], [2]. Body ownership specifically, which refers to the sense that a part of one’s body belongs to oneself, has been studied as a fundamental aspect of bodily self-consciousness [3], [4]. Moreover, body ownership and aspects of bodily self-awareness influence clinical outcomes because they can modulate the regulation of one’s physical self [5], [6], [7].

The rubber hand illusion (RHI) is an experimental paradigm that manipulates body ownership of the hand and is widely used to investigate the processes that underlie various aspects of bodily self-awareness [6], [8]. When participants watch a rubber hand being stroked by a paintbrush in synchrony with strokes applied to their own corresponding hand, they typically experience a change in body ownership such that the rubber hand “feels like one’s own hand” [9]. This illusory feeling of body ownership is thought to be associated with the multimodal integration of visual, tactile, and proprioceptive information in the brain [10]. When participants take ownership of the artificial counterpart, the real hand exhibits a decrease in skin temperature, slower processing of tactile information, and enhanced histamine reactivity, which suggests that a cortical body matrix integrates the perceptual and homeostatic regulation of the body [2], [6], [11]. The experience of the RHI is associated with in a distributed network in the brain [7]. The ventral premotor cortex has been suggested as a key brain structure for the multisensory representation of one’s own body during the RHI [12]. When the rubber hand is fully incorporated into the body, artificial limbs were able to evoke a similar level of activity in the insula and the anterior cingulate cortex, indicating that the bodily ownership of the rubber hand is associated with changes in the interoceptive system [13]. The experience of body ownership of the rubber hand as measured by the effect of multisensory integration and recalibration of hand position was positively correlated with activity in the right posterior insula [14].

Acupuncture, an ancient East Asian therapeutic technique, uses needles to stimulate a particular part of the body for the purpose of inducing beneficial effects during clinical treatment [15]. Acupuncture stimulation affects microcirculatory blood flow near the inserted needle as well as regional blood flow in various organs [16], [17]. Neuroimaging studies investigating acupuncture have observed overlapping brain responses in a number of cortical and subcortical brain regions. For example, activation has been observed in cortical sensorimotor and salient networks such as the insula, thalamus, anterior cingulate cortex, as well as primary and secondary somatosensory cortices, whereas deactivation has been identified in the limbic-paralimbic neocortical network in areas such as the medial prefrontal cortex, caudate, amygdala, posterior cingulate cortex, and parahippocampus [18], [19]. Accordingly, recent reviews of functional magnetic resonance imaging (fMRI) studies revealed common brain activations in the sensorimotor cortical network and common deactivations in the limbic-paralimbic-neocortical network following acupuncture needle stimulation [20], [21]. A number of convergent results and promising new data provide some pointers for understanding the neural mechanisms of acupuncture therapy in the context of the modern medical mainstream [20], [21]. Despite recent progress, however, some questions still remain controversial and a) Are specific traditional acupuncture points related to their specifically claimed functions? (Question of Point-specificity) [19], [22], [23], b) How is the acupuncture-specific sensation, called *DeQi* sensation, located functionally in the brain? [24], [25], [26], c) Are pain-related areas desensitized in the brain during or after acupuncture treatment, explaining the phenomenon of acupuncture analgesia? [27], [28], [29], [30], d) Can the specific (physiological) effects be distinguished with non-specific (psychological or placebo) effects of acupuncture? [15], [31], [32], [33]. e) What is the role of the interceptive attention system, such as somato-spatial attention, body awareness, and anticipation of stimulation in the physiological responses to acupuncture? [34], [35] The answers to these questions have been somewhat contradictory, and left mostly controversial.

From the perspective of cognitive neuroscience, acupuncture is not only seen as a ‘simple needling’ but also as a complex treatment comprised of multimodal sensory stimulation that interacts with various factors, including bodily self-awareness [36], [37]. The role of psychosocial and contextual factors, including body image and body schema, are thought to exert an important influence on the clinical effects of acupuncture [38]. Neuroimaging studies have suggested that the insula, which is known to modulate the interoceptive system, plays an important role in the effects of acupuncture [35], [39]. The insula was activated to a greater extent during real acupuncture than during the placebo intervention in patients with osteoarthritis [39]. Considering a salient component of acupuncture analgesia, focused attention and accentuated bodily awareness on the specific body part with acupuncture-induced sensation could contribute to the top-down modulation of nociceptive afference and the central pain matrix [35]. Conversely, we can assume that acupuncture might not properly exert its influence under reduced bodily awareness. Thus, it is conceived that components of this bodily self-awareness can be involved in the modulation of physiological responses to acupuncture stimulation. In the current study, we hypothesized that modification of body ownership can reduce the neurophysiological responses to acupuncture stimulation, especially through the disruption of the interoceptive system in the brain.

The present study aimed to investigate whether modifications of body ownership would result in measurable physiological changes in response to acupuncture stimulation using LASER Doppler and fMRI, respectively.

## Methods and Materials

### Procedures for measuring changes in local blood flow (Experiment 1)

#### Participants

Advertisements were used to recruit a total of 28 right-handed participants (age: 22.8±0.5 years; 15 males and 13 females) for this study from the general population of students, staff, and visitors to Kyung Hee University in Seoul, Republic of Korea. Participants with no history of neurological, psychiatric, or visual disorders were included in this study. Handedness was self-reported by the participants in a screening questionnaire prior to the experiment, and participants were prohibited from drinking alcohol or caffeine or taking any drugs or medications on the day of the experiment. Each participant received a detailed explanation of the study, and written informed consent was obtained prior to participation. All experiments in this study were conducted in accordance with the guidelines issued by the human subjects committee and approved by the Institutional Review Board of Korea University in Seoul, Republic of Korea.

#### Experimental design

**Figure 1:**
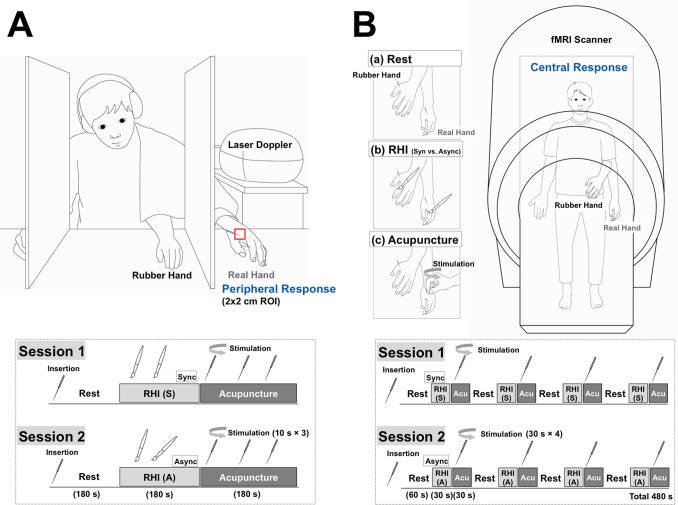
Experimental procedures. A: Procedures for peripheral responses (Experiment 1). Two small paintbrushes stroked the rubber hand (strokes were approximately 2 cm in length) and the hidden real left hand of the participant at a frequency of 1 Hz for 180 seconds as synchronously as possible under one condition (synchronous condition: S) and asynchronously under the other condition (asynchronous condition: A); RHI: Rubber hand illusion induction. Participants received acupuncture needle stimulation at a frequency of 1 Hz for 10 seconds every 1 minute during the 3-minute acupuncture stimulation period on the hidden (real) left hand immediately after commencing the RHI under the synchronous or asynchronous condition (Acu: Acupuncture stimulation). B: Procedures for brain responses (Experiment 2). There were two randomized sessions: a synchronous condition (S) and an asynchronous condition (A). The acupuncture needle was stimulated at the LI4 acupoint in the left hand out of sight of the participant according to the beats of a 1-Hz metronome transmitted via earphones (Acu: Acupuncture stimulation). One session included a four-block tactile-stimulation condition (30 seconds) for the RHI and a four-block acupuncture stimulation (30 seconds); these were performed successively. Tactile stimulation was synchronously or asynchronously applied with short strokes from two brushes (approximately 2 cm in length) at a frequency of 1 Hz during the tactile-stimulation block; RHI: Rubber hand illusion induction.

The setup followed standard procedure and was designed and executed almost identically to a previous study [9], placing a rubber hand in front of each participant while their left hand was hidden from sight. The experiments followed a within-subjects cross-over design in which the independent variable was a synchronous versus an asynchronous brush touch on the hand to measure illusory body ownership. In both the synchronous and asynchronous sessions, participants received 180 seconds of acupuncture needle stimulation on the hidden (real) left hand immediately after the RHI (Fig. 1A). The order was randomized such that participants were randomly assigned to one of two groups (i.e., they received either the synchronous or the asynchronous session first). The interval between the two sessions was approximately 2 days.

#### Tactile stimulation for the induction of the rubber hand illusion

Participants were told to fixate on the rubber hand (Korean Prosthetic Limbs Research Institute; Seoul, Korea), to not look elsewhere, and to not move any of their fingers during the experiment. Two small paintbrushes were used to stroke both the rubber hand and the participant’s hidden real left hand (strokes were approximately 2 cm in length) at a frequency of 1 Hz for 180 sec. This was done synchronously under one condition (synchronous condition) and asynchronously under the other condition (asynchronous condition).

#### Acupuncture stimulation

First, participants were informed that physiological data, including LASER Doppler measurements, would be recorded while they received acupuncture stimulation. Then, each participant was led to the experimental room and seated with their left and right arms on a table in the same manner as in a standard RHI experiment [9]. At the beginning of the session, an acupuncture needle was inserted at acupoint LI4 on the dorsum of the left hand radial to the midpoint of the second metacarpal bone. The needles were 0.20 mm in diameter and 40 mm long (Seirin Acupuncture, Inc.; Kasei, Japan). Participants were instructed to look at the rubber hand during the acupuncture stimulation.

After the tactile stimulation on the rubber hand and the real hand, standard acupuncture needle stimulation (the same as in clinical acupuncture treatments) was applied by a licensed and experienced Doctor of Korean Medicine, which consisted of rotating the needle at a frequency of 1 Hz for the duration of 10 seconds every 1 minute. Therefore, the total duration of acupuncture insertion was 3 minutes, including a total of 30 seconds (3×10 seconds) of acupuncture needle manipulation.

To confirm that the synchronous and the asynchronous sessions did not differ in terms of manual acupuncture stimulation, Acusensor2 force sensors (Stromatec Inc, Burlington, VT, USA) were attached to the needle handle to measure the degree of stimulation (rotation frequency) and the biomechanical force (torque amplitude: rotation force) [40]. The rotation frequency and torque amplitude were calculated over a period of 10 seconds based on raw data. During needle rotation, the rotation frequency was calculated using fast Fourier analyses of the raw data by Data Viewer software (Stromatec Inc.), and the torque amplitude of peaks was calculated manually as the mean value.

#### LASER Doppler measurement

Measurements of skin blood perfusion around the site of acupuncture stimulation (acupoint LI4) were performed using a LASER Doppler perfusion imager (LDPR; PeriScan PIM III system; Perimed AB, Sweden). Before the tactile stimulation for the induction of the RHI, the real hand (left hand) of participants was immobilized with a cylindrical object to ensure positioning, and measurements were carried out every 5 seconds for a total duration of 180 seconds. The temperature in the laboratory room was kept consistent and controlled (24–26°C) throughout the experiment. The LASER Doppler scanning was conducted on the participants’ real hand out of sight without any sound and did not produce any sensation. Peripheral blood flow (PBF; symbolized as R*_i_,*_[*i* = 1, 2, 3, …, 36]_) was defined as the mean blood flux in each scan (5 seconds) around the LI4 acupoint (2×2 cm), and the change in PBF was calculated using the following formula: (R*_i_*-R_1_)/R_1_. For assessing the responses during and after acupuncture stimulation, each 5 sec was subtracted from a 10 s baseline prior to the initial acupuncture stimulation (onset) and averaged across the 180 s of each session, resulting in change in scores that reflected increases from the baseline.

#### Rubber hand illusion and self-reported DeQi sensation rating

Right after the completion of tactile stimulation on the rubber hand and real hand (synchronous and asynchronous), participants reported their perception of the RHI by answering the most relevant question (Q3) on the RHI Perception Scale: “I felt as if the rubber hand were my hand” [9]. Acupuncture is generally accompanied by the perception of a complex set of sensations, called “*DeQi*” including aching, dull, heavy, numb, radiating, spreading and tingling [41]. It is considered to be associated with acupuncture treatment outcomes [42]. After each acupuncture stimulation session, participants evaluated their acupuncture-induced *DeQi* sensation, including heaviness, soreness, and numbness, using a 100-mm visual analogue scale [43].

#### Data analysis

All values are expressed as means ± standard errors. RHI ratings, subjective pain ratings, and PBF changes under the synchronous brush-stroking (induction of illusory body ownership) and the asynchronous brush-stroking (control) sessions were compared using paired *t*-tests. The level of significance was set at 0.05 for all analyses. Statistical analyses were performed using the Statistical Package for Social Sciences for Windows 20.0 (SPSS Inc.; Chicago, IL, USA).

### Procedures for measuring brain responses with fMRI (Experiment 2)

#### Participants

A total of 17 right-handed participants (age: 23.0±0.8 years; 10 males and 7 females) were recruited by advertisement from the general population of students, staff, and visitors to Kyung Hee University in Seoul, Republic of Korea for participation in this study. Participants with no history of neurological, psychiatric, or visual disorders were included in this study. Handedness was self-reported by the participants in a screening questionnaire prior to the experiment, and participants were prohibited from drinking alcohol or caffeine or taking any drugs or medications on the day of the experiment. Each participant received a detailed explanation of the study and written informed consent was obtained prior to participation. All experiments in this study were conducted in accordance with the guidelines issued by the human subjects committee and approved by the Institutional Review Board of Korea University in Seoul, Republic of Korea.

#### Experimental design

During the brain scans, participants lay in a supine position on the MRI table, and their left hand was comfortably placed on the MRI table while the rubber hand was placed 15 cm above their left hand so that their real hand was hidden under the rubber hand. The rubber hand was orientated in an anatomically plausible position, pointing slightly right toward the midline of the body (20°–30°). The right hand was also comfortably placed on the MRI table. A mirror attached to the head coil enabled participants to look at the rubber hand; the position of the rubber hand and/or the head-coil mirror was adjusted to ensure a clear view of the rubber hand. Before the start of scanning, the acupuncture needle was inserted at the LI4 acupoint (on the dorsum of radial to the midpoint of the second metacarpal bone) both in the left rubber hand and left real hand out of sight of the participant. Prior to scanning, we ensured that none of the participants was able to see their own hand. The two sessions (synchronous and asynchronous conditions) were conducted in a random order. Each session included four blocks of tactile stimulation (30 seconds) to invoke the RHI and four subsequent blocks of acupuncture stimulation (30 seconds) immediately after. A total of 240 volumes were acquired during each session for 480 seconds (Fig. 1B).

#### Induction of the rubber hand illusion and acupuncture stimulation

The participants were told to look at the rubber hand (Korean Prosthetic Limbs Research Institute), to not look elsewhere, and to not move any of their fingers during the experiment. Two small paintbrushes (approximately 2 cm in length) were used to stroke the rubber hand and the hidden real left hand of the participant at a frequency of 1 Hz for 180 seconds. This was done synchronously under one condition (synchronous condition) and asynchronously under the other condition (asynchronous condition).

Acupuncture stimulation was always applied at acupoint LI4 on the left hand by a licensed and experienced Doctor of Korean Medicine using non-magnetic titanium sterile acupuncture needles that were 40 mm long and 0.20 mm in diameter (DongBang Acupuncture Inc.; Boryeoung, Republic of Korea). All stimulations were administered according to the beat of a 1-Hz metronome transmitted via earphones. Following each trial, subjects were asked about their experience of the illusion via headphones in the scanner.

#### fMRI data acquisition

The fMRI scans were acquired with a MAGNETOM Trio 3 T scanner (Siemens, Erlangen, Germany) using echo planar imaging (EPI) with a 64×64 matrix (TE = 30 ms, TR = 2000 ms) across 37 slices with a thickness of 4 mm. To minimize movement artifacts, the head of each participant was fixed using a head holder. All scans were acquired by a well-trained professional operator. Each scan session contained 210 volumes of the whole brain in a 37 axial slice acquisition (TR = 2000 ms, TE = 30 ms, flip angle = 90°, field of view = 240×240 mm^2^ , voxel size = 3.8×3.8×4.0 mm^3^ ). As an anatomical reference, a 3-dimensional T1-weighted magnetization-prepared rapid gradient echo (MPRAGE) image data set was acquired using the following parameters: TR = 2000 ms, TE = 2.37 ms, flip angle = 9°, field of view = 240×240 mm^2^ , voxel size = 0.9×0.9×1.0 mm^3^ , 192 slices.

#### fMRI data analysis

Analysis of the fMRI data was performed using Statistical Parametric Mapping software (SPM8; Wellcome Institute of Imaging Neuroscience; London, UK) implemented in Matlab 7.1 (Mathworks Inc.; Natick, MA). The data were realigned and co-registered on a mean image, normalized to a template, and smoothed with an 8-mm full-width-at-half-maximum (FWHM) Gaussian kernel. The model was estimated by applying individual movement regressors.

The first four volumes of each session were discarded to allow for T1 equilibration. All the remaining functional images were corrected for slice acquisition timing and head motion. For each participant, the functional images were realigned to the first task-relevant image to correct for head motion and were co-registered to the structural T_1_-weighted anatomical images. To decrease spatial noise, the images were spatially normalized using the Montreal Neurological Institute (MNI) template, resampled with an isotropic 2 mm×2 mm×2 mm voxel size, and smoothed with an 8-mm FWHM isotropic Gaussian kernel.

Each acupuncture stimulation was modeled as a boxcar function convolved with a canonical hemodynamic response function that began at the onset of each stimulation. Contrast maps were generated for brain reactivity to acupuncture stimulation, and the individual contrast images in each session were then included in a second-level random-effects analysis. Brain responses to acupuncture stimulation were recorded under both synchronous and asynchronous conditions. For statistical inference, a threshold of *P*<0.05 was used following a correction for multiple comparisons (family-wise error corrections, FWE). To plot the regions of brain activation associated with acupuncture stimulation, the neuronal activity in specific brain areas was correlated over time by extracting the averaged percent signal change in regions of interests (ROIs) separately in the common brain areas (i.e. insula and secondary somatosensory cortex (SII)). The anatomical ROIs for insula and SII were obtained using the Wake-Forest University PickAtlas tool box version 2.4 (WFU PickAtlas; [44]. Signal changes were extracted using the MarsBaR ROI toolbox for SPM8 (http://marsbar.sourceforge.net/).

A multiple regression analysis was conducted to evaluate the association of the contrast images to acupuncture stimulation between asynchronous and synchronous session with individual ratings as two separate covariate vectors (1) the differences of RHI score (synchronous – asynchronous session) and (2) the differences of *DeQi* score (asynchronous – synchronous session). For the regression analysis, activation was considered significant at p<0.001 (uncorrected) with a minimum cluster extent threshold of 10 contiguous voxels.

#### Rubber hand illusion and self-reported DeQi sensation ratings

After finishing each session (synchronous and asynchronous), participants reported their perception of the RHI by answering question 3 on the RHI Perception Scale: “I felt as if the rubber hand were my hand” [9]. The participants also filled out the acupuncture-induced *DeQi* sensation questionnaire using a seven-point Likert scale ranging from “strongly disagree” (−3) to “strongly agree” (+3).

#### Data analysis

All values are expressed as means ± standard errors. RHI ratings, subjective *DeQi* ratings, and BOLD signal changes were compared between the synchronous and asynchronous sessions using paired *t*-tests. The level of significance was set at 0.05 for all analyses. Statistical analyses were performed using the Statistical Package for Social Sciences for Windows 20.0 (SPSS Inc.).

## Results

### Experiment 1

#### Changes in local blood flow following acupuncture stimulation during the RHI

**Figure 2:**
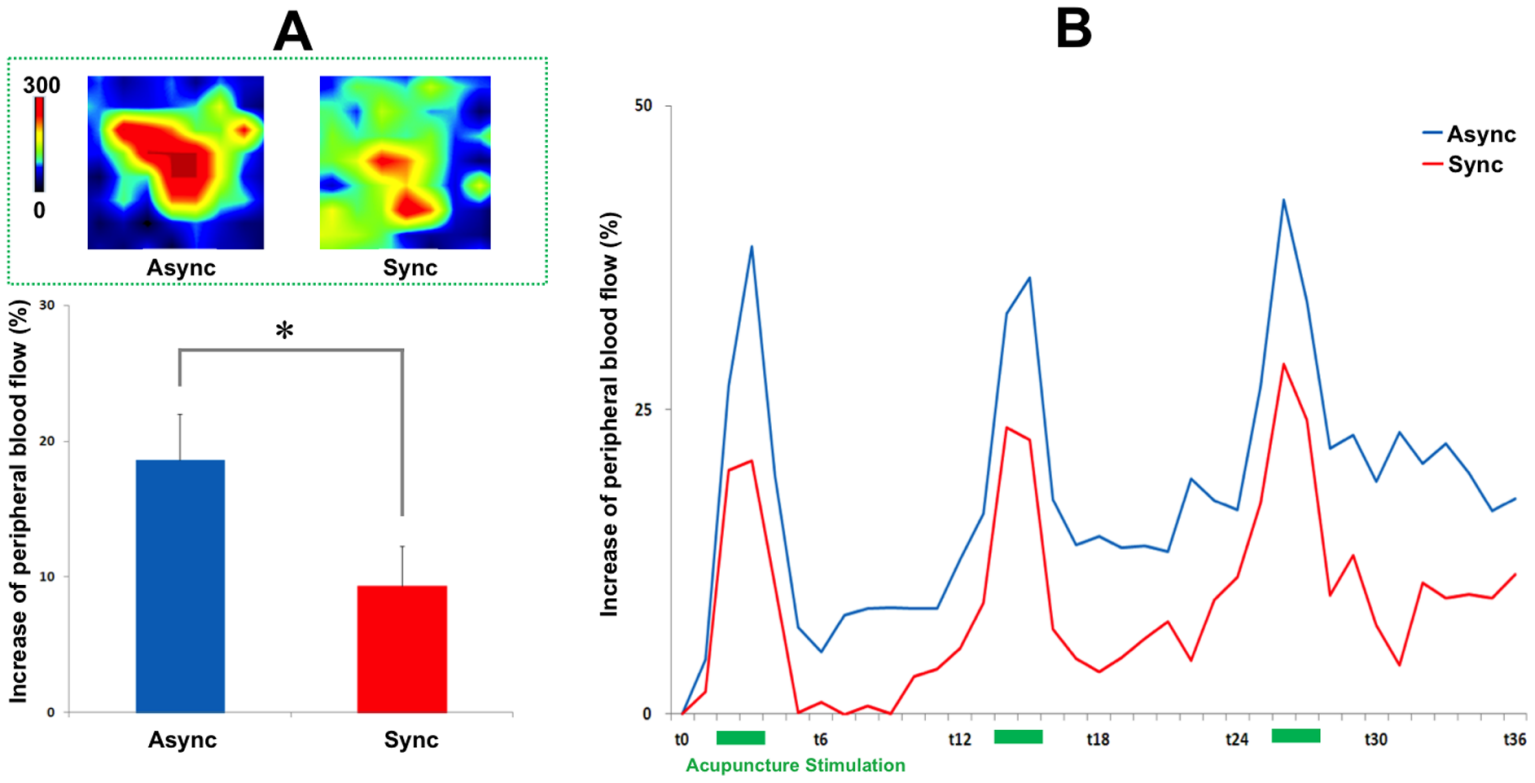
Peripheral responses to acupuncture stimulation during the RHI. A representative example of the peripheral responses to acupuncture stimulation around the LI4 acupoint (2×2 cm) is shown in upper column A. The increase in peripheral responses to acupuncture stimulation was significantly different between the synchronous and asynchronous brush-stroke sessions, as seen in lower column A (18.6±3.3 vs. 9.3±2.8%, *t* = 2.544, *P*<0.05). Peripheral responses to acupuncture stimulation are presented as the mean peripheral blood-flow change over time. Values are means ± standard errors.

Local blood flow measurements are presented as the mean PBF change over time. A significant difference was observed in the increase in local blood-flow change following acupuncture stimulation when the asynchronous and synchronous brush-stroking sessions were compared (18.6±3.3 vs. 9.3±2.8%, *t* = 2.544, *P*<0.05; Fig. 2). No significant differences were observed between the asynchronous and synchronous brush-stroke sessions in rotation frequency (degree of stimulation) or torque amplitude (biomechanical force) (frequency: 1.3±0.1 vs. 1.3±0.1 Hz, *t* = −0.414, *P*>0.684; torque: 15.4±5.0 vs. 13.8±4.2 µNm, *t* = −0.295 *P*>0.771, respectively).

#### Perception of the rubber hand illusion and self-reported DeQi sensation rating

We found a significant difference between the asynchronous and synchronous brush-stroke sessions in the self-reported perception of the RHI (−1.0±0.3 vs. 1.5±0.2, *t* = 8.520, *P*<0.001). No significant differences were observed in self-reported *DeQi* sensations when the asynchronous and synchronous brush-stroking sessions were compared (heaviness: 3.6±0.6 vs. 3.1±0.5, *t* = 0.769 *P*>0.449; soreness: 3.4±0.5 vs. 3.7±0.6, *t* = 0.403 *P*>0.690; numbness: 3.8±0.6 vs. 3.6±0.6, *t* = 0.256 *P*>0.800).

### Experiment 2

#### Brain responses to acupuncture stimulation during the rubber hand illusion

**Figure 3:**
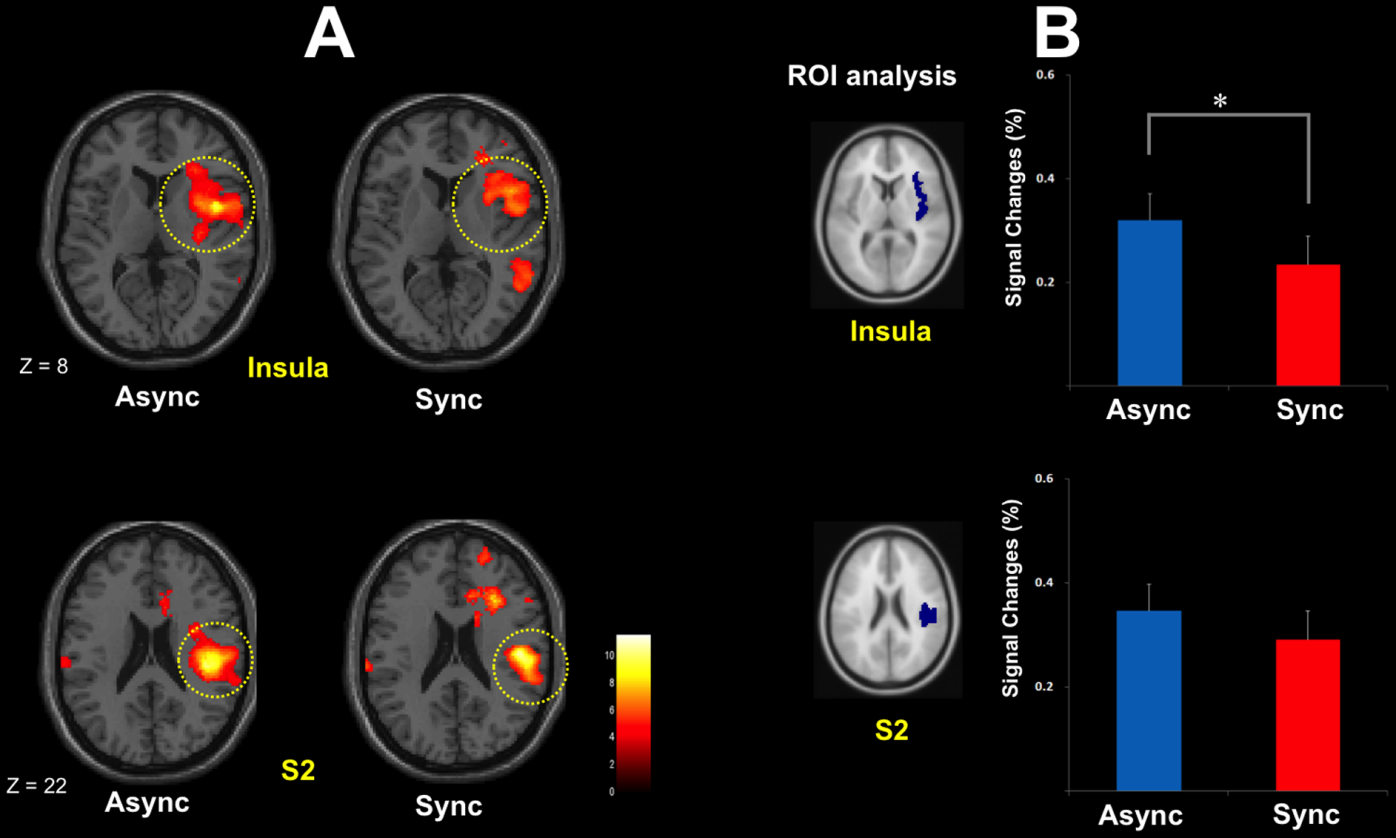
Brain activations in response to acupuncture stimulation. A: Brain activations in response to acupuncture stimulations were observed in the contralateral secondary somatosensory cortex (SII) and insula under both the asynchronous (right SII: 44, −26, 26; *t = *11.52; *Z = *5.9; right insula: 44, 2, 8; *t = *9.78; *Z = *5.5) and synchronous (right SII: 50, −20, 20; *t = *9.33; *Z = *5.39; right insula: 56, 20, 0; *t = *8.9; *Z = *5.27) conditions. All two peaks were *p*<0.05, corrected, and all coordinates are in MNI space. B: To plot the regions of brain activation involved in acupuncture stimulation, the averaged percent signal change in anatomical regions of interests (ROIs), including the right insula and SII, were extracted. When the acupuncture stimulation occurred, BOLD responses in the right insula (0.32±0.04 vs. 0.23±0.05%, *t* = 2.517, *P*<0.05; A) but not the SII (0.34±0.05 vs. 0.29±0.05%, *t* = 1.413, *P*>0.177; B) differed significantly under the asynchronous and synchronous sessions.

Brain activations following acupuncture stimulation were observed in the contralateral secondary somatosensory cortex (SII) and insula under both the asynchronous (right SII: 44, −26, 26; *t = *11.52; *Z = *5.9; right insula: 44, 2, 8; *t = *9.78; *Z = *5.5) and synchronous (right SII: 50, −20, 20; *t = *9.33; *Z = *5.39; right insula: 56, 20, 0; *t = *8.9; *Z = *5.27) sessions (Table 1). All peaks are *p*<0.05, corrected, and all coordinates are in MNI space (Fig. 3A).

**Table 1:** Activated brain regions to acupuncture stimulation in asynchronous session and synchronous session (n = 17).

Activated regions	MNI Coordinates			Peak *t*	*p* value (FWE corrected)	Cluster size
	X	Y				
Async						
(R) Insula	44	2	8	9.78	<0.05	106
(R) SII	44	−26	26	11.52	<0.05	200
Sync						
(R) Insula	56	20	0	8.9	<0.05	36
(R) SII	50	−20	20	9.33	<0.05	59

Comparison of asynchronous and synchronous sessions revealed that the brain responses following acupuncture stimulation were significantly altered in the insula during the RHI. Differences in signal change (less brain activation during the synchronous sessions) were significant in the right insula (0.32±0.04 vs. 0.23±0.05%, *t* = 2.517, *P*<0.05) but not in SII (0.34±0.05 versus 0.29±0.05%, *t* = 1.413, *P*>0.177; Fig. 3B).

#### Perception of the rubber hand illusion and self-reported DeQi sensation rating

In Experiment 2, a significant difference between the asynchronous and synchronous brush-stroking sessions was observed in the self-reported perception of the RHI (−0.6±0.4 vs. 0.9±0.3, *t* = 4.622, *P*<0.001). No significant differences were observed in self-reported *DeQi* sensation when comparing the asynchronous and synchronous brush-stroke sessions (1.6±0.4 vs. 2.2±0.2, *t* = 1.830, *P*>0.086).

#### Individual differences in rubber hand illusion score and *DeQi* score co-varied with brain activations to acupuncture stimulation during rubber hand illusion

**Figure 4:**
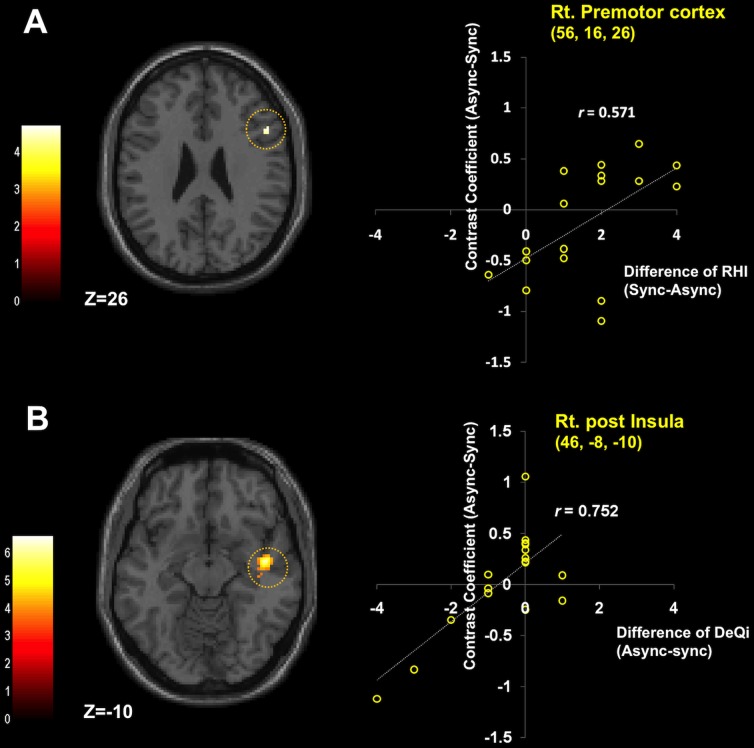
Individual differences in brain activations to acupuncture stimulation. A: Correlation between rubber hand illusion score and brain activations to acupuncture stimulation during reduced body ownership. Normalized SPM T-maps overlaid on the corresponding axial T1-weighted images showing statistically significant (p<0.001, uncorrected with 10 continuous voxels) brain activation correlations between the rubber hand illusion (synchronous vs. asynchronous session) and brain responses to acupuncture stimulation in asynchronous condition compared to synchronous condition. Brain activation was observed in the right ventral premotor cortex (x = 52, y = 16, z = 26, Z = 3.34, *r = *0.571, *p*<0.05). B: Correlation between *DeQi* score and brain activations to acupuncture stimulation during reduced body ownership. Brain activation related to *DeQi* score (asynchronous vs. synchronous session) was observed in the right posterior insula (x = 46, y = −8, z = −10, Z = 4.38, *r* = 0.752, *p*<0.001).

We determined whether individual differences in RHI score (synchronous vs. asynchronous session) co-varied with functional brain activity responses to acupuncture stimulation (asynchronous vs. synchronous session). Higher RHI scores demonstrated significant co-variation with activities in the right ventral premotor cortex (x = 52, y = 16, z = 26, Z = 3.34, *r = *0.571, *p*<0.05, Fig. 4A).

We also determined whether individual differences in *DeQi* score (asynchronous vs. synchronous session) co-varied with functional brain activity responses to acupuncture stimulation (asynchronous vs. synchronous session). Higher *DeQi* scores demonstrated significant co-variation with activities in the right posterior insula (x = 46, y = −8, z = −10, Z = 4.38, *r* = 0.752, *p*<0.001, Fig. 4B).

## Discussion

The current study demonstrated that a disrupted sense of body ownership induces significant changes in both local PBF and brain BOLD responses during acupuncture needle stimulation. Experiment 1 used LASER Doppler technology and found that the degree of increased blood flow around the site of acupuncture manipulation was significantly decreased when disembodiment (disrupted sense of body ownership) was induced in the same hand receiving the acupuncture stimulation. Experiment 2 used fMRI and found that brain activations following acupuncture stimulation were significantly reduced in the right insula when disembodiment was induced in the hand receiving the acupuncture stimulation. These findings suggest that body ownership is a crucial factor that influences physiological responses during acupuncture stimulation.

In Experiment 1, when body ownership and bodily self-awareness were preserved in a normal way (asynchronous session), an increase in PBF was observed during acupuncture stimulation (Fig. 2). These findings are consistent with previous studies that reported enhanced microcirculatory blood flow near the acupoints following acupuncture stimulation [16], [17]. In contrast, when body ownership was disrupted (synchronous sessions), a significant decrease in peripheral microcirculation was observed around acupoint LI4 relative to asynchronous session. These findings indicate that the peripheral physiological responses following acupuncture stimulation are influenced by modifications of body ownership, at least around the site of acupuncture stimulation (left hand). To assure that the differences in PBF following acupuncture stimulation were not derived from discrepancies in acupuncture manipulations, the degree of and the biomechanical force behind the acupuncture manipulation were controlled for with force sensors. In our experiment, an experimenter conducted acupuncture manipulation immediately after he stroke both the rubber hand and the hidden real hand. In order to minimize a possible confounding factor in which a non-blinded acupuncturist could be biased to conduct acupuncture differently between the synchronous and the asynchronous sessions, we tried to make the acupuncturists conduct the manipulation using similar rotation frequencies, torques, and amplitudes during the acupuncture manipulation. The sensor measurements confirmed that there was no significant difference of the degree of stimulation (rotation frequency) and the biomechanical force (torque amplitude: rotation force) between the two sessions. Although unlikely given the professional training of the acupuncturist, we cannot exclude other possible bias to the outcome, such as tone of voice.

In Experiment 2, brain activations in the right insula and SII were observed in all sessions in which acupuncture stimulation was applied, whereas significant differences in BOLD signal change were observed only in the right insula when comparing synchronous and asynchronous brush-stroke sessions (Fig. 3). The common brain activations in the insula and SII in the both asynchronous and synchronous session were generally consistent with previous studies, which found that acupuncture stimulation leads to enhanced sensorimotor cortical network [20]. The observation of less activity in the right insula following acupuncture stimulation during the induction of the RHI (synchronous sessions) is noteworthy. The insula is known to generally modulate awareness of pain, temperature, sensual touch, and other bodily feelings using cutaneous mechanoreceptive stimuli [45]. It has often been suggested that the insular cortex is a unique neural substrate that instantiates subjective feelings from the body and feelings of emotion in the immediate present, which is highlighted by the central role of this area in “interoception” and “awareness of bodily signals” [46].

With the multiple regression analyses, we demonstrated differences in neural substrates for subjective ratings from the RHI and the *DeQi* sensation. Firstly, the RHI scores significantly correlated with the brain activations to acupuncture stimulation (asynchronous vs. synchronous session) in the ventral premotor cortex (Fig. 4A). Ventral premotor cortex has been shown to play a crucial role in multisensory integration in the context of the RHI [12], [47]. Previous studies have demonstrated that neural activity in the premotor cortex was correlated with the strength of illusory perceptions during the RHI [12], and damages in fibers connecting the ventral premotor cortex with other brain regions impaired the occurrence of RHI sensations [47]. It is therefore likely that the different brain responses in premotor cortex responding to the acupuncture stimulation between asynchronous and synchronous condition were much more associated with the degrees of the RHI across the subjects. Secondly, the difference of *DeQi* sensation significantly correlated with the brain activations to acupuncture stimulation (asynchronous vs. synchronous session) in the posterior insula in the current study (Fig. 4B). The insula, as a key structure of the interoceptive system, has been studied in relation to acupuncture treatment under some conditions [35], [39]. It is assumed that the differential brain responses in the posterior insula in response to acupuncture stimulation between the two sessions were reflected in the substantial variability between subjects in the change of *DeQi* sensation.

The phenomenal incorporation of the rubber hand is also reflected by brain activation in the insula, which can be explained by its association with the basic processes of self-consciousness [14]. Previous fMRI and positron emission tomography (PET) studies have observed brain activations in the insula when comparing BOLD responses during real acupuncture and sham (placebo) acupuncture stimulation. This suggests that enhanced bodily attention following acupuncture may operate as a therapeutic mechanism under some conditions [35], [39]. Taken together, these findings indicate that bodily self-awareness plays a central role during acupuncture treatment and that modifications of body ownership may disturb the function of acupuncture as a somatosensory-guided mind-body therapy via modulation of the interoceptive system that is represented by the insula. Further studies using sham-acupuncture are needed to address this issue in detail.

Our results suggest important implications for acupuncture research. In East Asian cultures, acupuncture stimulation is frequently employed for various clinical treatments, and the concept of *Qi* is often used to describe a distinct kind of bodily perception that explains how people feel, control, and experience their own sense of self. Achievement of appropriate *DeQi* sensation is considered to be vital for acupuncture treatment. It is thought that such perceived needling-induced sensations not only direct patients’ attention to the specific points on the body, but that they also modulate cognitive and affective aspects [20], [38]. In fact, certain components ascribed to the *DeQi* sensation, or perceived *Qi* during acupuncture treatment, may share the characteristics of particular aspects of “bodily self-awareness” because feeling and the perception of oneself are crucial components of both. Recently, aspects of “bodily awareness” have been increasingly attracting the interest of researchers in many disciplines [48]. Studies in the fields of neuroscience and neurology have shown that the conscious sense of one’s physical self is closely linked to the physiological regulation of one’s physical self [3], [7]. It has also been shown that any disruption in the awareness of one’s physical self can have an important impact on the physiological regulation of the self [6]. It has been suggested that a body matrix, or a multi-sensory representation of peri-personal space and the space directly around the body, serves to maintain the integrity of the body at both the homeostatic and psychological levels and allows for the adaptation of our body structure and orientation to changes in the environment [2]. Findings demonstrating that components of this bodily self-awareness are also involved in the modulation of the clinical effects of acupuncture may have important implications for acupuncture research. Mohan et al. have failed to demonstrate that the RHI modulate pain threshold or pain evoked by individually calibrated high and low painful stimuli delivered on the real arm [49]. Our previous study also found no significant differences in self-reported pain rating to acupuncture stimulation between the asynchronous and the synchronous session [36]. In the current study, we found no significant differences of subjective *DeQi* ratings between the two sessions. Even though there were two different characteristics of acupuncture sensations, such as the pain domain and *DeQi* domain [41], our findings are partially supporting the previous findings that there were no differences of pain ratings during the RHI [36], [49]. Although we did not find significant differences of subjective *DeQi* ratings between the two sessions, differences of brain activations to acupuncture stimulation in the posterior insula was associated with the difference of *DeQi* sensation *between the two sessions* in the current study. It is believed that the insula plays a crucial role in the enhanced body awareness on the specific body site by acupuncture evoked-*DeQi* sensation. Further studies are needed to determine whether reduced embodiment around the acupuncture points during RHI can affect the *DeQi* sensation and/or clinical outcome.

Still, our study possesses several limitations. First, as we did not implement a 2×2 (Body ownership×Intervention) factorial design using a tactile control (such as a non-penetrating placebo needle), we cannot claim that bodily self-awareness is a crucial factor influencing physiological responses that is restricted only to acupuncture stimulation. The rationale for not conducting such a control lies in the difficulty of providing non-penetrating sham acupuncture. Such needles are commonly used as a placebo control for acupuncture experiments [50]. In our previous study, we demonstrated specific patterns of brain activations related to genuine acupuncture compared to sham acupuncture, i.e. non-penetrating placebo needles [15], [31]. As such a needle relies on the visual impression that a real needle is being inserted to the skin, however, researchers have claimed that its validity is limited when using such placebo acupuncture out of sight of the participant [51]. Given that participants in the present study can only see the rubber hand in front of them and are not able to see their own hand, a non-penetrating placebo needle would not work well as a placebo control stimulation. Further studies are necessary in order to explore the role of body ownership specific to acupuncture stimulation when compared to other types of tactile stimulation. Second, the acupuncture needle was inserted into only the real hand in experiment 1. In order to minimize visual differences between the rubber hand and the real hand, on the other hand, we performed experiment 2 with the acupuncture needle inserted into the real hand as well as in the rubber hand. In our previous study, the sympathetic activations to acupuncture stimulation in the real hand were markedly higher during the synchronous session compared to that of the asynchronous session only when a visual expectation of the acupuncture needle stimulation existed in the rubber hand [36]. In this case, it is assumed that the participants seemed to have already allowed the incorporation of the artificial body part into their self-representation, and can experience an additional “visual capture” of the acupuncture needling. Future research will be needed to investigate the role of the visual factor of the acupuncture needle in the rubber hand during reduced body ownership. Last but not at least, we did not include a control condition in which a rubber hand was not displayed. It is well established that some participants can still feel a sense of embodiment or ownership towards the rubber hand even without any touch. In order to minimize other confounding factor, such as touch, we were trying to compare the physiological changes of acupuncture between asynchronous and synchronous session in this study. As we did not examine the physiological responses to acupuncture in a third condition without the rubber hand, however, we cannot explicitly analyze the role of the visual factor provided by the artificial hand in our experiments.

The current study demonstrates that both local blood flow changes and brain responses following acupuncture needle stimulation are altered by modifications of body ownership. When body ownership was disrupted by the RHI during acupuncture treatment, local blood flow to the site of acupuncture stimulation was reduced and brain activations during acupuncture stimulation were significantly decreased in the insula, a key region involved in the interoceptive system. Acupuncture can be regarded as a complex treatment comprised of multimodal sensory stimulation that interacts with various factors, including bodily self-awareness [36], [37]. These findings suggest that physiological responses during acupuncture stimulation can be influenced by the modification of body ownership. Further research is necessary to ascertain whether the clinical effects of acupuncture are associated with enhanced bodily self-awareness and, if so, how strongly.
